# Neuropathology of SCA34 showing widespread oligodendroglial pathology with vacuolar white matter degeneration: a case study

**DOI:** 10.1186/s40478-021-01272-w

**Published:** 2021-10-24

**Authors:** Kokoro Ozaki, Takashi Irioka, Toshiki Uchihara, Akane Yamada, Ayako Nakamura, Takamasa Majima, Susumu Igarashi, Hiroshi Shintaku, Mayumi Yakeishi, Yukio Tsuura, Yasushi Okazaki, Kinya Ishikawa, Takanori Yokota

**Affiliations:** 1 Department of Neurology and Neurological Science, Graduate School of Medical and Dental Sciences, Tokyo Medical and Dental University, 1-5-45 Yushima, Bunkyo-ku, Tokyo, Japan; 2 Laboratory for Comprehensive Genomic Analysis, RIKEN Center for Integrative Medical Sciences, 1-7-22 Suehiro-cho, Tsurumi-ku, Yokohama, Kanagawa Japan; 3 Diagnostics and Therapeutics of Intractable Diseases, Graduate School of Medicine, Intractable Disease Research Center, Juntendo University, 2-1-1 Hongo, Bunkyo-ku, Tokyo, Japan; 4 Department of Neurology, Yokosuka Kyosai Hospital, 1-16 Yonegahama dori, Yokosuka, Kanagawa Japan; 5 Division of Surgical Pathology, Tokyo Medical and Dental University Hospital, 1-5-45 Yushima, Bunkyo-ku, Tokyo, Japan; 6 Departments of Diagnostic Pathology and Clinical Laboratory, Yokosuka Kyosai Hospital, 1-16 Yonegahama dori, Yokosuka, Kanagawa Japan; 7 The Center for Personalized Medicine for Healthy Aging, Tokyo Medical and Dental University, 1-5-45 Yushima, Bunkyo, Tokyo Japan

**Keywords:** Spinocerebellar ataxia type 34 (SCA34), Neuropathology, Fatty acid elongase, Very long-chain fatty acids (VLCFA), Tauopathy, Four-repeat tau, Tuft shaped astrocytes, Progressive supranuclear palsy (PSP)

## Abstract

Spinocerebellar ataxia type 34 (SCA34) is an autosomal dominant inherited ataxia due to mutations in *ELOVL4*, which encodes one of the very long-chain fatty acid elongases. SCA38, another spinocerebellar ataxia, is caused by mutations in *ELOVL5*, a gene encoding another elongase. However, there have been no previous studies describing the neuropathology of either SCA34 or 38. This report describes the neuropathological findings of an 83-year-old man with SCA34 carrying a pathological *ELOVL4* mutation (NM_022726, c.736T>G, p.W246G). Macroscopic findings include atrophies in the pontine base, cerebellum, and cerebral cortices. Microscopically, marked neuronal and pontocerebellar fiber loss was observed in the pontine base. In addition, in the pontine base, accumulation of CD68-positive macrophages laden with periodic acid-Schiff (PAS)-positive material was observed. Many vacuolar lesions were found in the white matter of the cerebral hemispheres and, to a lesser extent, in the brainstem and spinal cord white matter. Immunohistological examination and ultrastructural observations with an electron microscope suggest that these vacuolar lesions are remnants of degenerated oligodendrocytes. Electron microscopy also revealed myelin sheath destruction. Unexpectedly, aggregation of the four-repeat tau was observed in a spatial pattern reminiscent of progressive supranuclear palsy. The tau lesions included glial fibrillary tangles resembling tuft-shaped astrocytes and neurofibrillary tangles and pretangles. This is the first report to illustrate that a heterozygous missense mutation in *ELOVL4* leads to neuronal loss accompanied by macrophages laden with PAS-positive material in the pontine base and oligodendroglial degeneration leading to widespread vacuoles in the white matter in SCA34.

**Supplementary Information:**

The online version contains supplementary material available at 10.1186/s40478-021-01272-w.

## Introduction

Spinocerebellar ataxias (SCAs) are a group of autosomal dominant disorders that typically show progressive ataxic symptoms, including truncal and limb ataxias and dysarthria. Patients with SCAs exhibit a variety of neurological symptoms and signs, including oculomotor disturbances, pyramidal and extrapyramidal tract signs, and autonomic failures. Currently, SCA1–48 have been described and over 30 causative genes have been identified [18]. Recent reports show that mutations in the ELOVL fatty acid elongases 4 and 5 genes (*ELOVL4* and *ELOVL5*) cause SCA34 (OMIM #133,190) and SCA38 (#615,957), respectively [12, 15, 19, 35]. The prevalence of SCA34 is rare [36] but SCA34 patients from the USA, Brazil, Canada, and Japan, have been identified [8, 9, 12, 19, 35, 36, 47]. To date, five heterozygous mutations (c.504G>C, p.L168F; c.736T>G, p.W246G; c.512T>C, p.I171T; c.539A>C, p.Q180P; c.698C>T, p.T233M in *ELOVL4*) causing SCA34 have been reported. Prototypal manifestations of SCA34 include slowly progressive gait ataxia with erythrokeratodermia skin lesions, which are observed in patients with the mutations c.504G>C, p.L168F; c.539A>C, p.Q180P; c.698C>T, p.T233M. Patients with SCA34 show a broad spectrum of signs and symptoms, including pyramidal and extrapyramidal tract signs, autonomic dysfunction, peripheral neuropathy, and possible cognitive decline and psychomotor symptoms [4, 19, 35, 36]. A recent report on SCA34 pedigree with c.512T>C, p.I171T mutation indicated retinal degeneration [47]. As described in our previous study of Japanese pedigrees with c.736T>G, p.W246G mutation, patients with the original mutation c.504G>C, p.L168F in Canada also displayed pontine hot cross bun sign hyperintensity on brain magnetic resonance imaging (MRI) [4].

*ELOVL4* mutations are known to cause allelic diseases. For example, mutations leading to carboxy-terminal truncations of *ELOVL4* cause autosomal dominant Stargardt macular dystrophy type 3 [49]. Ichthyosis, spastic quadriplegia, and mental retardation (ISQMR) is a recessive disease caused by nonsense and frameshift mutations in *ELOVL4* [3].

*ELOVL4* encodes the 314 amino acid multipass transmembrane protein, ELOVL4. ELOVL4 is expressed in the retina, skin, brain, testis, and other organs [1] and is mainly localized in the endoplasmic reticulum. ELOVL4 is a key enzymatic component in the elongation of saturated and unsaturated very long-chain (C26 to C28 or longer) fatty acids. Of the seven elongases (ELOVL1 to ELOVL7) in humans, ELOVL4 plays an important role in the synthesis of fatty acids with the longest carbon chains, which can be over C30. The elongated fatty acids are utilized for the synthesis of important lipids, such as sphingomyelin, ceramide, phosphatidylcholine, and cholesterol esters [1, 25]. Repertoires of fatty acids synthesized by the ELOVL4 enzyme and the complex lipids which contain the synthesized fatty acids, differ in a tissue-specific manner. And, different mutations in *ELOVL4* may preferentially affect the synthesis of various lipids. For example, an experimental study using knock-in rats with p.W246G mutation indicated preferential disturbance in synthesizing very long-chain saturated fatty acids [2]. In the lipid-rich human brain, lipid substances, including phospholipids, glycolipids, and cholesterol, account for 70% of the dry weight of the myelin sheath surrounding axons [26]. Thus, myelin may be affected by *ELOVL4* mutations in SCA34. In animal models, very long-chain saturated fatty acids synthesized by ELOVL4 are enriched in neuronal synaptic vesicles and are involved in the modulation of presynaptic release [14, 21]. However, no autopsy studies and, thus, no information on the neuropathological consequences of *ELOVL4* mutations in humans with SCA34 have been reported. The mechanisms leading to neurodegeneration and subsequent neurological signs and symptoms in humans are not known.

For the first time, we describe the autopsy of a patient with SCA34, with a known c.736T>G, p.W246G (NM_022726.4) mutation in *ELOVL4*. Neuropathological findings include marked neuronal loss and pontocerebellar fiber degeneration in the pontine base, CD68-positive macrophages laden with periodic acid-Schiff (PAS) and Gallyas-positive material, and oligodendrocyte and myelin degeneration with frequent cerebral white matter vacuoles. Furthermore, four-repeat tauopathy with neurofibrillary tangles and glial fibrillary tangles was unexpectedly observed in a spatial pattern compatible with progressive supranuclear palsy (PSP). The glial fibrillary tangles include those which resembled tuft-shaped astrocytes, which is a distinctive feature of PSP. These findings demonstrate that a heterozygous *ELOVL4* missense mutation can cause various distinct neuropathological features of neuronal and oligodendroglial degeneration. The four-repeat tauopathy compatible with PSP may be an incidental co-occurrence or one of the neuropathological consequences of the *ELOVL4* mutation in SCA34.

## Case presentation

We studied an 83-year-old man who presented with slowly progressive gait ataxia at the age of 46 years old. He was an index case from our previous report of a Japanese pedigree with SCA34 [patient II-1 in family A] caused by a heterozygous c.736T>G, p.W246G mutation in *ELOVL4* [35]. As previously described, the patient had ophthalmoplegia more obvious in the vertical direction than the horizontal direction, nystagmus, dysarthria, pyramidal tract signs, truncal and limb ataxia, trivial akinesia, and autonomic symptoms, including bladder disturbance and constipation [35]. The patient had been wheelchair bound since his sixties and had a gastrostomy due to dysphagia. The brain MRI showed marked atrophy in the pontine base and cerebellum, hot cross bun sign, mild atrophy in the cerebral cortices, and incidental small infarcts in the basal ganglia, as described in our previous report [35]. The patient had recurrent urinary tract infections and cellulitis. He had gastric carcinoma, urinary tract carcinoma, and subsequent urinary tract obstructions. The patient succumbed to death due to postrenal kidney failure at the age of 83. He was autopsied at a postmortem interval of 8 h 34 min, after obtaining written consent from his family.

### Methods for neuropathological analyses

This study was conducted with permission from the institutional review boards of Tokyo Medical and Dental University, Japan (G2000-198). Written consent was obtained from his family for this study. The whole brain and spinal cord were fixed in formalin. Samples from the left side of the supratentorial regions and bilateral infratentorial regions of the brain were analyzed, including the superior and middle frontal gyrus, pre- and post-central gyrus, mammillary body and basal nucleus of the Meynert, superior temporal gyrus, temporal pole, hippocampus, amygdala, caudate nucleus, putamen, globus pallidus, thalamus, visual cortex, cerebellar cortices, and deep cerebellum. In addition, the whole brainstem, cervical and lumbar spinal cord, and pituitary were analyzed. Sections (5 μm) of paraffin-embedded samples were subjected to Hematoxylin–Eosin (HE), Klüver-Barrera (KB), and PAS staining and immunohistochemistry. Antibodies against Neurofilament H phosphorylated (1:5000, mouse monoclonal, SMI-31, BioLegend, USA, RRID:AB_10124140), glial fibrillary acidic protein (1:1000, rabbit polyclonal, DAKO, USA, RRID:AB_10013382), myelin basic protein (MBP) (1:1000, rabbit polyclonal, IBL, Japan, RRID not available/IBL Cat # 16,141), Olig2 (1:1000, rabbit polyclonal, IBL, Japan, RRID not available/IBL Cat # 18,953), CD68 (1:1000, clone KP1, mouse monoclonal, DAKO, USA, RRID:AB_2314148), Iba1 (1:500, rabbit polyclonal, WAKO, Japan, AB_839504), ubiquitin (1:1000, rabbit polyclonal, DAKO, USA, RRID:AB_2315524), RD3 (3-repeat tau) (1:3000, mouse monoclonal, MILLIPORE, USA, RRID:AB_310013), RD4 (4-repeat tau) (1:1000, mouse monoclonal, MILLIPORE, USA, RRID:AB_310014), alpha-synuclein (1:10,000, mouse monoclonal clone 5G4, Roboscreen, Germany, RRID not available/ Order #847–0,102,004,001), and tau (tau2) (1:1000, clone TAU-2, mouse monoclonal, Sigma, USA, RRID:AB_477581) were used. Free-floating sections from formalin-fixed samples were subsequently stained with oil-red, Sudan black, and OsO4 for light microscopy, and anti-Neurofilament H phosphorylated antibody (SMI-31), 4′,6-diamidino-2-phenylindole (DAPI), and fluoroMyelin (1:300, Invitrogen, USA) for immunofluorescence microscopy. Light microscopes (NanoZoomer S210, Hamamatsu Photonics, Japan and BX-7/DP-5, Olympus, Japan) and a confocal immunofluorescence microscope (A1R, Nikon Corp, Japan) were used for observation and digital recording of the prepared samples.

For electron microscopy, a small part of the formalin-fixed corpus callosum at the coronal section at the mammillary body level was excised, fixed in 2% glutaraldehyde buffer, and post-fixed in 1% OsO_4_. The section was dehydrated and embedded in Epon. Ultrathin sections were made and stained with uranyl acetate and lead citrate. Sections were examined with a transmission electron microscope (H-7100/XR41, Hitachi, Japan). We examined the variant table from exome-sequencing in this patient [35], and checked for any mutations in genes *MAPT*, *PRKN*, *TARDBP*, and *LRRK2*.

### General pathology findings

General autopsy findings included urothelial carcinoma of the urinary bladder, which invaded the prostate gland with no metastases to other organs, early-stage carcinoma of the stomach (tubular adenocarcinoma), pyelonephritis, bilateral postrenal renal failure, and atherosclerosis of the coronary artery and abdominal aorta. No evidence of metastases was found.

### Neuropathological findings

**Fig. 1:**
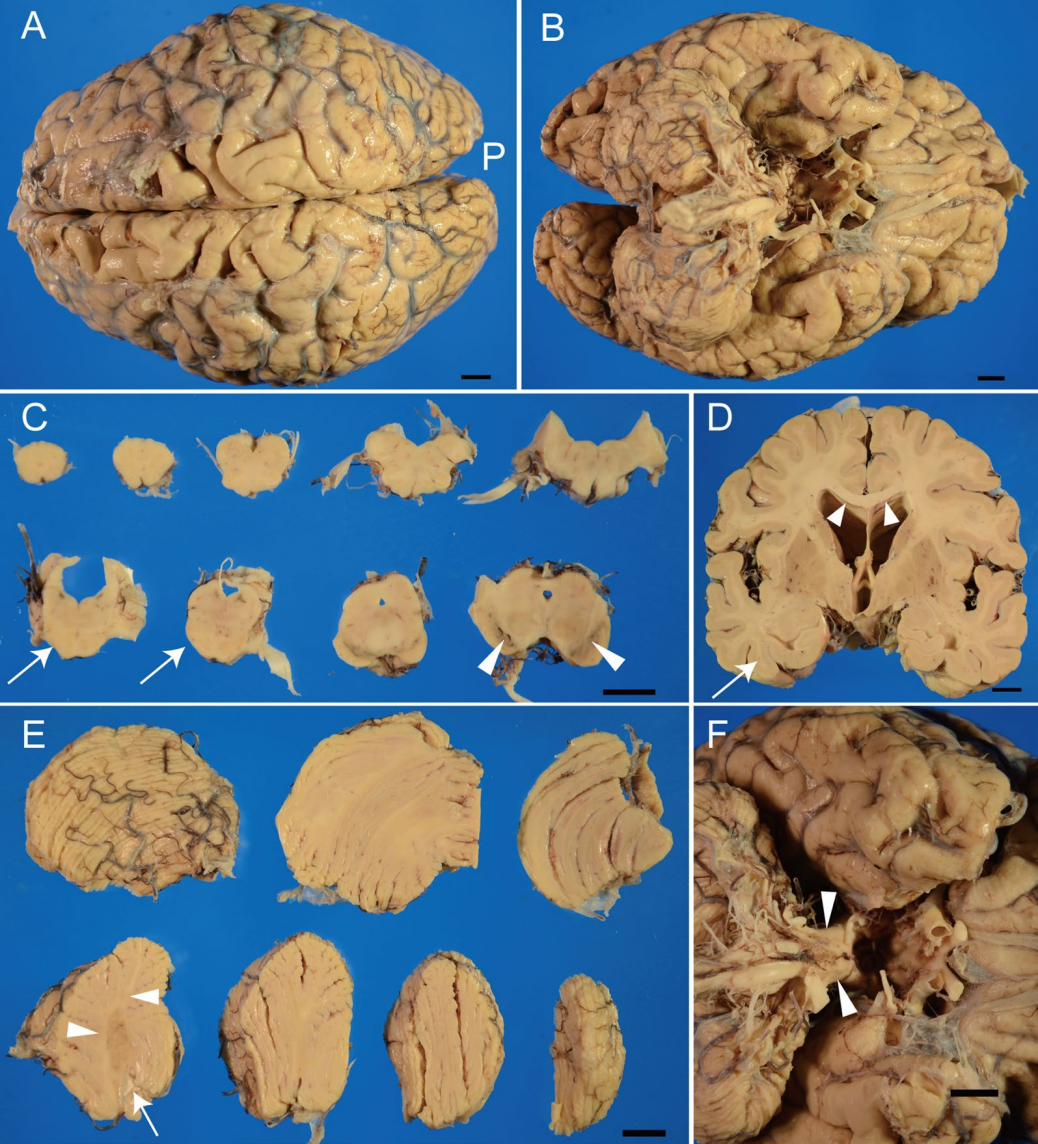
Macroscopic views of the brain of an autopsy case with SCA34. (**a** and **b)** Superior and inferior views, respectively, of brain surfaces. P denotes posterior pole to show direction. Atrophy of cerebral cortices, especially in the medial temporal lobes, was noted (see also **d**). (**c**) Transverse sections of the brainstem, from the medulla oblongata to the midbrain. White arrows indicate severe atrophy in the pontine base. In addition, the ventral parts of the midbrain and medulla were more atrophied than the dorsal parts. Depigmentation of the substantia nigra was also noted (arrowheads). (**d**) Coronal section of the cerebrum showed largely preserved caudate nucleus, putamen, and globus pallidus. Arrowheads indicates moderately atrophied corpus callosum. Mild atrophy of the cerebral white matter was observed (arrow). (**e**) Horizontal sections of right cerebellar hemisphere and sagittal sections of left hemisphere. Arrowheads show atrophied white matter. Arrow indicates obscured hilum of dentate nucleus. (**f**) Inferior view of the brain shows marked atrophy of the basis pontis (arrowheads). Scale bar = 1 cm (**a** to **f**)

Total brain weight was 974 g (990.7 g after formalin fixation, the brainstem and cerebellum weighed 11.2 and 71.8 g, respectively). Macroscopic observation revealed marked atrophy in the brainstem, especially in the pontine base. The ventral parts of the midbrain and medulla were more atrophied than the dorsal parts. The bilateral crus cerebri were remarkably atrophic and the pyramis in the medulla was barely detected (Fig. 1c). In addition, cerebral hemispheres predominantly in the bilateral medial temporal lobes and cerebellum were atrophied (Fig. 1a, b, e, and f).

Moderate atrophy was observed in the corpus callosum, while mild atrophy was found in the cerebral white matter (Fig. 1d). The caudate nucleus, globus pallidus, and amygdale were macroscopically normal. A small infarction in the left putamen was detected (not shown). The basal ganglia were otherwise unremarkable.

The midbrain was atrophic, especially in the cerebral peduncles, with mild enlargement of the aqueduct. Depigmentation was found in the substantia nigra (Fig. 1c). Superior cerebellar peduncles and the red nucleus were unremarkable. Remarkable atrophy was found in the pons, especially in the pontine base, and in the middle cerebellar peduncles (Fig. 1c). The inferior olivary nuclei were preserved. Despite the marked atrophy of the cerebellar white matter, the cerebellar folia and the dentate nuclei were relatively preserved (Fig. 1e). The spinal cord and roots were not atrophic.

### Microscopic observations

The histological findings can be summarized in the following three changes.

**Fig. 2:**
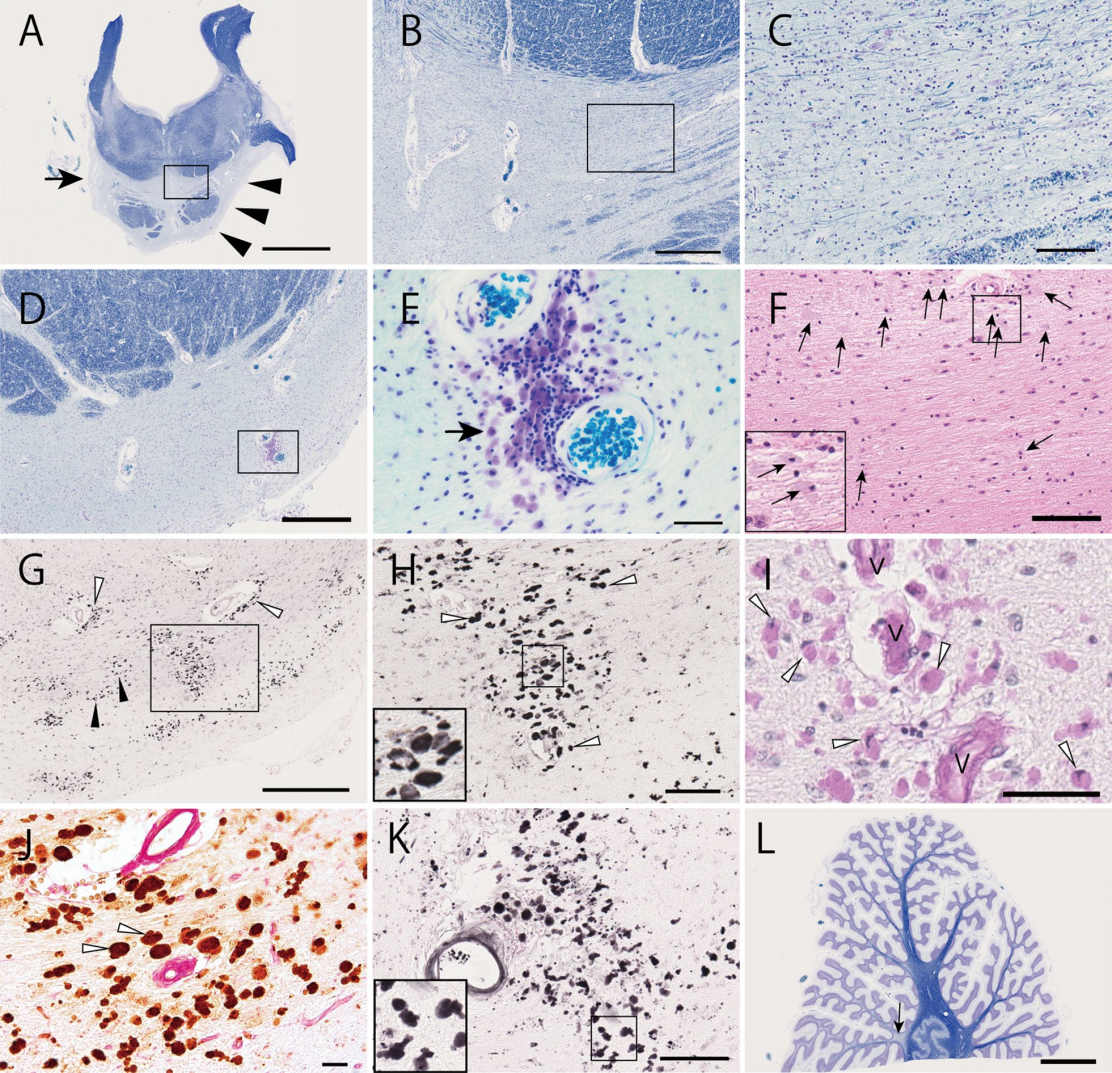
Microscopic observation of the pons and cerebellum from an autopsy case with SCA34. **a** Semi-macroscopic view of the pons after Klüver-Barrera (KB) staining. Arrowheads indicate severe atrophy of the pontine base. An arrow indicates demyelination of the transverse fibers. **b** Transverse fibers between the medial lemniscus and pyramidal tract showed severe degeneration (magnified from the rectangle area in **a**). **c** Further magnified view of the transverse fibers between the medial lemniscus and pyramidal tract (magnified from the rectangle area in B). Very scarce and degenerated fibers were observed. **d**, **e**, and **f** After KB (**d** and **e**) and Hematoxylin–Eosin (HE) (**f**) staining, the most ventral part of the pontine base also showed severe degeneration of pontocerebellar fibers and neuronal loss. Cells with irregular shaped dense nuclei and violet cytoplasm after KB staining as shown in **e** (magnified from the rectangle area in **d**) or pale gray after HE staining (arrows in **f** and magnified in its inset) were observed. **g** and **h** immunostaining for CD68. Strongly CD68-positive cells were widespread (white and black arrowheads in **g**) in the pontine base, excluding the pyramidal tracts. These cells occasionally accumulated around the vasculature (white arrowheads in **g** and **h**). **h** is a magnified view from the rectangle in **g**. The inset in H illustrates a magnified view from the rectangular area in the center. **i** The cytoplasm of the CD68-positive cells was positively stained with periodic acid-Schiff (PAS) (arrowheads). V denotes vessels. **j** Double staining with PAS (red) and anti-CD68 (brown) illustrates co-staining for PAS and CD68 in the same cells (arrowheads). The double-staining is shown as a dark-brown color, except for wall of the vasculature, which only stained for PAS. **k** These macrophages were positive for Gallyas silver impregnation method. **L** Semi-macroscopic view of the cerebellum showing white matter atrophy and mild and uneven demyelination (arrow). Scale bar = 5 mm (**a** and **l**), 500 μm (**b**, **d**, and **g**), 100 μm (**c**, **f**, **h**, and **k**), 50 μm (**e** and **l**), and 20 μm (**j**)

**Fig. 3:**
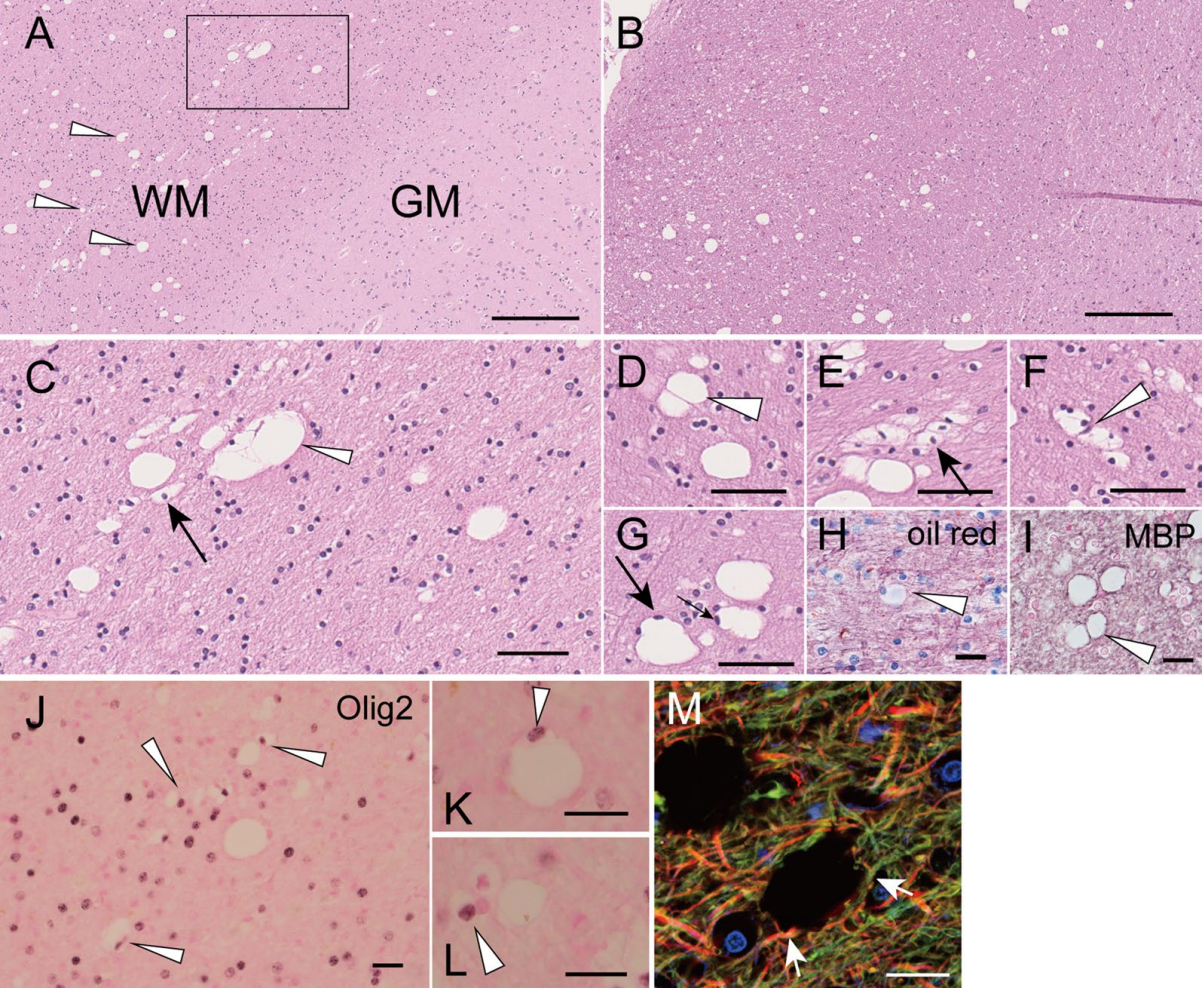
Light microscopic observation of white matter vacuoles in an autopsy case with SCA34. **a** Hematoxylin–Eosin (HE) staining of the frontal cortex showing vacuoles in the white matter (arrowheads) but few in the gray matter. WM and GM denote white and gray matter, respectively. **b** Vacuoles in the right superior cerebellar peduncle are shown. **c** A magnified view showing single vacuoles and adhered multiple vacuoles (arrowhead) (magnified from the rectangular area in **a**). Cells with swollen cytoplasm and round and bright nuclei, often accompanied by one or more similar adjacent cells (arrows in **c** and **e**), suggest that these vacuoles were derived from oligodendrocytes. **d**, **e**, **f**, and **g** Various forms of vacuoles in the cerebral white matter were observed. A thin septum was often seen between adjacent vacuoles (arrowheads in **d** and **f**). In some vacuoles, flattened nuclei were observed in the rim (arrows in **g**). **h** Oil red staining did not reveal lipid substance in vacuoles (arrowhead). **i**, Anti-MBP staining of thin rims and septum, suggesting that components of myelin surround the vacuoles (arrowhead). **j**, **k**, **l** Nuclei inside the rims of vacuoles were often Olig2-positive. **m** Confocal immunofluorescence microscopy using anti-Neurofilament H antibody (axonal neuronal protein, in red), fluoroMyelin (staining lipid contents of myelin, in green), and 4′,6-diamidino-2-phenylindole (DAPI) (nuclear staining, in blue). Arrows indicate the neuronal fibers, including myelin circumscribing a vacuole and an oligodendrocyte. Scale bar = 250 μm (**a** and **b**), 50 μm (**c** to **g**), 20 μm (**h** to **m**)

**Fig. 4:**
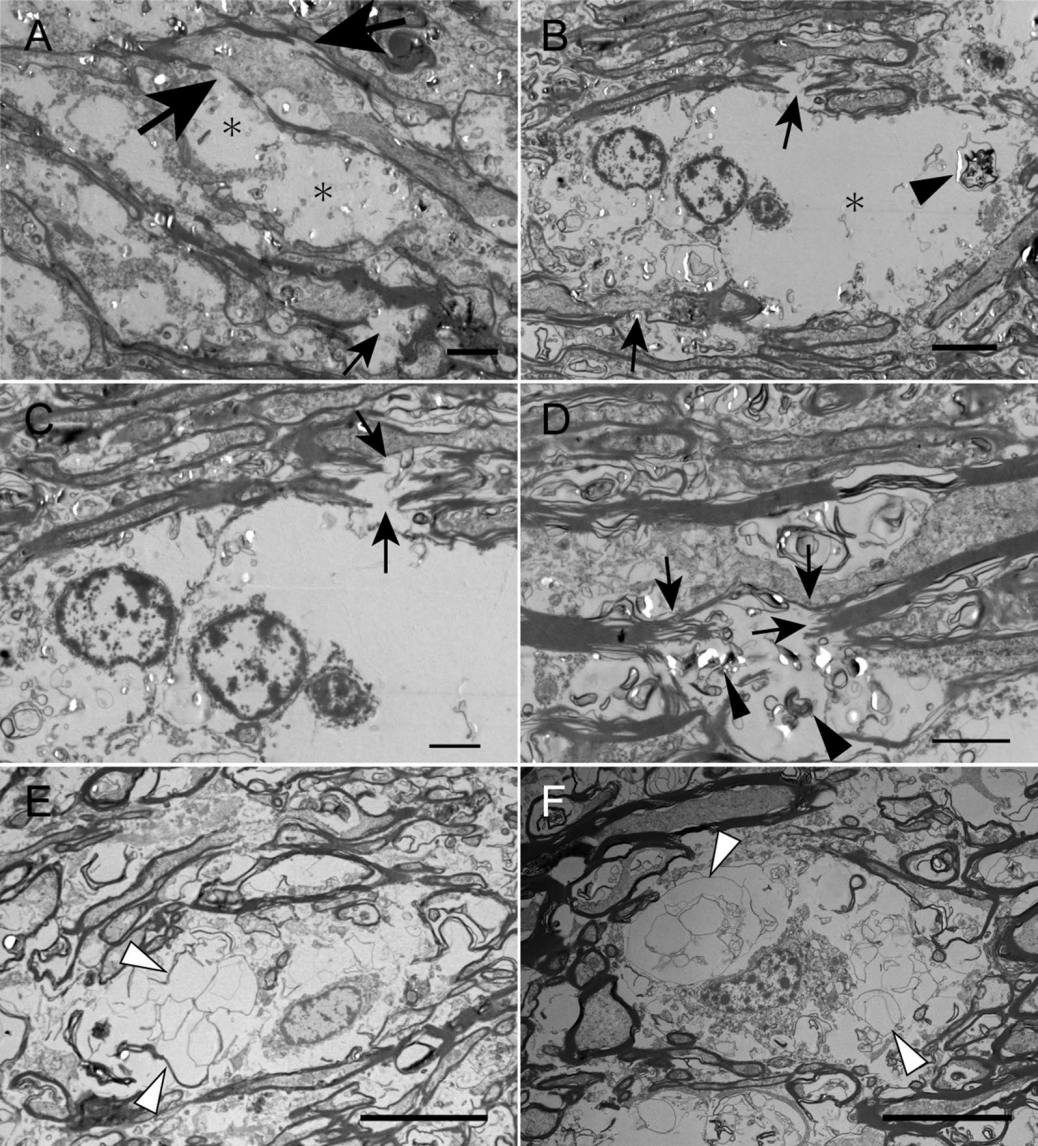
Electron microscopic observation of white matter abnormalities in an autopsy case with SCA34. **a**, **b**, **c**, **d**, **e**, and **f**, transmission electron microscopy of the corpus callosum. **a** Vacuoles (asterisks) associated with discontinuous and degenerated myelin, which ran nearby (large and small arrows, respectively), are shown. **b** A ballooned cytoplasmic space (asterisk) and nucleus are at the outermost space of the oligodendroglial cytoplasm. Arrows indicate deformed myelin. Myelin digestion is shown (arrowhead). **c** An enlarged view from the same site as B showing disrupted myelin fibers (arrows). **d** Deformed and disrupted myelin (arrows) accompanying cytoplasmic digestion of myelin (arrowheads) is shown. **e** and **f** Loose membrane structures, which likely reflect disrupted myelin, in swollen oligodendrocytes, can be observed (arrowheads). Scale bar = 10 μm (**e** and **f**), 4 μm (**a** and **b**), 2 μm (**c** and **d**)

**Fig. 5:**
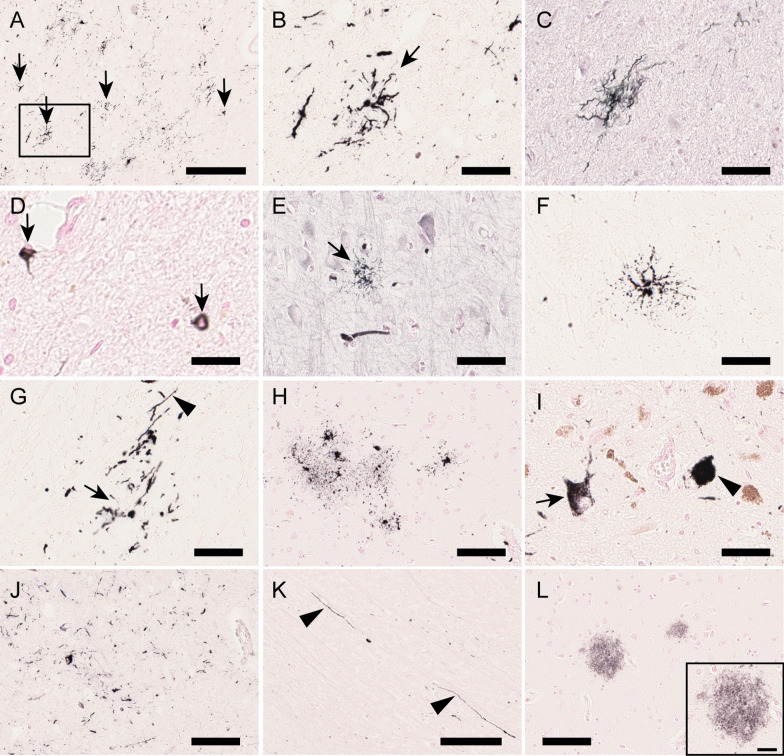
Tauopathy in SCA34. **a** and **b** The subthalamic nucleus showing frequent glial fibrillary tangles (arrows) stained with anti-four-repeat tau antibody. Magnified view (**b**) from the rectangular area in **a** showing some of the tangles that resemble a tuft-shaped astrocyte (arrow). **C** A tuft-shaped astrocyte exhibiting argyrophilia on Gallyas silver impregnation in the subthalamic nucleus. **d** Coiled bodies (arrows) in the subthalamic nucleus stained with the anti-phosphorylated tau antibody, AT8. **e** A tuft-shaped astrocyte (arrow) in the prefrontal cortex exhibiting argyrophilia on Gallyas silver impregnation. **f** A tuft-shaped astrocyte in the putamen stained with anti-four-repeat tau antibody. **g** Glial fibrillary tangles akin to a tuft-shaped astrocyte (arrow) and thread (arrowhead) in the external segment of the globus pallidus, stained with anti-four-repeat tau antibody. **h** Glial fibrillary tangles in the frontal cortex stained with anti-phosphorylated tau antibody AT8. **i** Pretangle (arrow) and neurofibrillary tangle (arrowhead) in the substantia nigra pars compacta stained with anti-phosphorylated tau antibody AT8. **j** The superior colliculus in the midbrain showing frequent glial fibrillary tangles stained with anti-four-repeat tau. **k** The intraaxial oculomotor nerve partly stained with anti-four-repeat tau antibody in the midbrain (arrowheads). **L** Few amyloid plaques stained with anti-amyloid antibody in the insular cortex (magnified view of a plaque in inset). Scale Bar = 250 μm (**a** and **k**), 100 μm (**h** and **l**), 50 μm (**b**, **c**, **e**, **f**, **g**, and **i**), 25 μm (**D** and inset in **L**)

1. Neuronal loss, pontocerebellar fiber loss, and CD68-positive macrophages laden with periodic acid-Schiff positive material in the pontine base
  Microscopic findings underlying the pontine base atrophy included marked neuronal loss in the pontine nucleus, destruction of transverse fibers, and gliosis (Fig. 2a to c). Cells with swollen cytoplasm (staining violet with KB) in the pontine base were observed. These cells were found in the pontine nucleus and transverse fibers and occasionally around vessels (Fig. 2d, e). These cells were CD68 positive and, thus, considered macrophages (Fig. 2g, h). The macrophages contained pale gray substances on HE-stained sections (Fig. 2f), which was positive for PAS (Fig. 2i). Double labeling for PAS and CD68 also supports this interpretation (Fig. 2j). Double staining for PAS and either Olig2 (see Additional file 1 Fig. 1A) or Iba1 (Additional file 1 Fig. 1B) indicated that these macrophages (CD68 positive cells) were not microglia or oligodendrocytes. These cells were glial fibrillary acidic protein–negative and, thus, were not astrocytes (Additional file 1 Fig. 1C). Accumulated PAS-positive substances suggested the presence of polysaccharides, such as glycogen, glycoproteins, or, most importantly, glycolipids.
  The macrophages in the pontine base unexpectedly exhibited argyrophilia on Gallyas silver impregnation method (Fig. 2k and Additional file 1 Fig. 1G and H). A previous report showed that loss of function for the yeast homolog of *ELOVL4* promoted alpha-synuclein aggregation in a yeast model of Parkinson’s disease [27]. We examined whether the mutation in *ELOVL4* led to alpha-synuclein aggregation in the current case with SCA34. Immunohistochemistry illustrated an absence of phosphorylated alpha-synuclein aggregation in the pontine macrophages, other pontine structures, basal ganglia, and motor cortex (Additional file 1 Fig. 1D, E, and F). Campbell–Switzer silver impregnation method, which can stain Alzheimer’s disease neurofibrillary tangles, senile plaques, and Lewy bodies [45], did not stain the pontine macrophages (Additional file 1 Fig. 1I). The macrophages did not exhibit positive staining for anti-three-repeat, four-repeat, or phosphorylated tau (Additional file 1 Fig. 1J, K, and L). Moderate but uneven tau2 antibody staining of the macrophages was observed (Additional file 1 Fig. 1M). The tau2 antibody presumably stains an atypical conformation of tau protein, for instances in a previous study, in microglia/macrophages of cerebral infarction or inflammation [44]. The CD68 positive macrophages laden with PAS-positive material were not observed in any brain structures other than the pontine base.
  In accordance with the severe degeneration of the pontine nucleus and pontocerebellar fibers, the cerebellar white matter was atrophied, especially in the terminal lobular areas compared to the deep cerebellar white matter (Fig. 2l). Purkinje cells were mildly to moderately reduced and atrophied (Additional file 1 Fig. 1N). Mild and uneven demyelination in the cerebellar white matter was also noted (arrow in Fig. 2l). The cerebellar cortices were relatively preserved compared to the white matter. The molecular layer was thin, while the granular layer was preserved.
2. Widespread vacuolar lesions and myelin degeneration consistent with oligodendrocyte degeneration
  Many vacuoles (Fig. 3a to m) were found in the cerebral white matter in the frontal, temporal, parietal, and posterior lobes, optic tract, corpus callosum, and internal capsule in HE stained sections. Vacuoles were also found along the intramedullary fibers of the ophthalmic nerve roots in the midbrain and the pontine tegmental area, including the medial lemniscus, superior cerebellar peduncle, trigeminal nerve root, and, to a lesser extent, the pyramidal tract (Fig. 3a, b, and Additional file 2 Fig. 2A to D). The vacuoles were notably absent in the atrophic pontocerebellar fibers of the pontine base, including the middle cerebellar peduncles. The vacuoles were rare in the anterior, lateral, and posterior columns of the spinal cord, but often observed along intramedullary fibers of anterior and posterior roots (Additional file 2 Fig. 2E to G).
  The vacuoles were mostly 15 to 50 μm in diameter in the paraffin-embedded sections and were very rare in gray matter throughout the brain. Solitary vacuoles and multiple adhered vacuoles were observed (Fig. 3c to g). The vacuoles did not appear to contain solid substances and were often associated with cells in a line harboring swollen clear cytoplasm and round nuclei (arrows in Fig. 3c and e). The nuclei remaining in these vacuoles or associated cells were often positive for Olig2, one of the oligodendroglial nuclear markers (Fig. 3j to l). Therefore, the vacuoles were likely derived from oligodendrocytes.
  The intravacuolar substances were negative for lipophilic staining, including oil red, Sudan-black, or OsO4 (Fig. 3h and Additional file 2 Fig. 2H and I). The thin lining of rims and septum of vacuoles were positive for MBP, suggesting components of myelin surround vacuoles (arrowhead in Fig. 3i). Confocal immunofluorescence microscopy using anti-Neurofilament H antibody, which stains the neuronal axons, in conjunction with fluoroMyelin, which stains the lipid contents of myelin, indicated that vacuoles displaced neural fibers, including myelin (arrows in Fig. 3m). Because MBP is one of the cytoplasmic components of myelin, these findings are compatible with the view that the vacuoles originated in oligodendroglia. Sections prepared using freezing methods from formalin-fixed tissues showed smaller vacuoles.
  To evaluate the ultrastructural features of vacuoles, oligodendrocytes, and myelin, we analyzed the corpus callosum, a part of the cerebral white matter, with electron microscopy. We found swollen oligodendrocytes with disrupted myelin (Fig. 4a to d); however, the large vacuolar lesions, which were observed using light microscopy, were rarely found in the electron microscopy. Loose membrane structures in swollen oligodendrocytes, which likely reflect disrupted myelin, were observed (arrowheads in Fig. 4e and f). These loose membranes were absent in paraffin-embedded sections, probably reflecting the fragility of the structures that were lost during processing. In summary, electron microscopy showed destructed myelin structures associated with swollen oligodendrocytes and degraded fine myelin inside the cells, which were not seen under the light microscope.
3. Tau accumulation in the brain of the SCA34 patient
  Because we found that PAS-positive macrophages of the pontine base were also positive for Gallyas silver impregnation but negative for alpha-synuclein accumulation, we next sought to evaluate the accumulation of tau protein. Tau is another major protein that can form pathologic aggregations exhibiting argyrophilia on Gallyas silver impregnation. The macrophages in the pontine base were negative for three- or four-repeat tau (3R tau and 4R tau) or phosphorylated tau (p-tau) staining, as described above; however, we unexpectedly found widespread neuronal and glial fibrillary tangles positive for phosphorylated tau in the brain, including the frontal cortex, subthalamic nucleus, putamen, globus pallidus, substantia nigra, and midbrain tegmentum, where four-repeat tau was dominant (see Table 1 and Fig. 5a to j).
  Morphological characteristics of the glial fibrillary tangles positive for four-repeat tau resembled tuft-shaped astrocytes, coiled bodies, and threads (Fig. 5b to g), which characterize progressive supranuclear palsy, while some tau-positive lesions were difficult to classify. In addition, we observed comparable three- and four-repeat tau positive neurofibrillary tangles in the parahippocampus, hippocampus, locus ceruleus, and, to a lesser extent, in the temporal cortex and amygdala (Braak neurofibrillary tangles (NFT) stage II) [10]. Trace amounts or no staining of senile plaque, which is suggestive of early-stage Alzheimer’s disease or possibly consistent with primary age-related tauopathy (PART) [13], were observed in these areas using anti-beta amyloid immunostaining (Fig. 5l). Part of the intraaxial fibers of the left oculomotor nerve was positive for anti-four-repeat tau staining (Fig. 5k).
4. Other microscopic observations of the SCA34 patient brain and spinal cord

**Table 1:** Distribution of neuronal loss, tauopathy, and macrophage accumulation in SCA34

Region	Neuronal loss	Neurofibrillary tangles and pretangles by AT8	Glial fibrillary tangles by AT8	Macrophage accumulation	Characteristic findings, mainly based on anti-three (3R) and four (4R) tau staining
Frontal cortex	−	±	+	−	Infrequent glial fibrillary tangles (GFTs) resembling tuft-shaped astrocytes (4R-tau positive). 4R-tau positive lesions (neuronal/glial) were more frequent than 3R (4R > 3R) as a whole
Primary motor cortex	−	±	+	−	4R > 3R. Rare GFTs resembling tuft-shaped astrocytes (4R-tau positive)
Temporal cortex (tip)	−	+	+	−	3R- and 4R-tau positive NFTs were both comparably observed (4R = 3R). 4R-tau positive astrocytes in subependymal regions of the lateral ventricule
Parietal cortex	−	±	±	−	4R > 3R
Posterior cortex (visual cortex)	−	−	±	−	
Caudate	−	−	±	−	
Putamen	−	±	+	−	GFTs resembling tuft-shaped astrocytes. 4R-tau positivity was much greater than 3R (4R > > 3R)
Globus pallidus	−	−	±	−	4R > > 3R
Subthalamic nucleus	±	+	+	−	4R > > 3R
Amygdala	−	+	+	−	4R = 3R
Parahippocampal cortex	−	+	−	−	4R = 3R
Hippocampus CA1/subiculum	−	+	−	−	4R = 3R
Hippocampus CA2-4	−	+	−	−	4R = 3R
Dentate gyrus	−	±	−	−	
Hypothalamus	−	±	±	−	
Thalamus	−	±	±	−	
Substantia nigra PR	−	±	−	−	
Substantia nigra PC	±	+	±	−	4R > > 3R
Midbrain tegmentum	−	±	±	−	4R > > 3R
Pontine nucleus	++	−	−	++	Marked macrophage accumulation in the pontine base
Locus ceruleus	±	+	−	−	4R = 3R
Cerebellar cortex	+	−	−	−	Purkinje cells were moderately decreased
Cerebellar nucleus (dentate)	−	±	±	−	4R > 3R
Inferior olive	−	−	±	−	
Dorsal column nuclei	±	−	−	−	
Spinal cord anterior horn	−	−	±	−	
Spinal cord dorsal horn	−	−	±	−	

− , negative; ± , trace; + , mild; ++ , severe. Tauopathy was evaluated by anti-phosphorylated tau (AT8) and anti-three-repeat and anti-four-repeat tau (3R and 4R, respectively) staining. Glial neurofibrillary tangles included those resembling tuft-shaped astrocytes, coiled bodies, threads, and other lesions which were difficult to classify

The volume of the substantia nigra pars compacta was decreased, while the substantia nigra pars reticulata was relatively preserved. Mild gliosis was observed in the red nuclei. The locus ceruleus nuclei were preserved but neurofibrillary tangles were observed, as described above. In the medulla oblongata, the inferior olivary nucleus, nuclei ambiguus, and spinal tract nucleus of the trigeminal nerve were preserved. In the spinal cord, motor neurons were spared.

## Discussion and conclusions

We present the first autopsy report of an SCA34 case caused by an *ELOVL4* mutation. The neuropathological findings included remarkable neuronal loss in the pontine nucleus, pontocerebellar fiber loss, pontine macrophages laden with PAS-positive material, widespread vacuoles in the white matter with oligodendroglial degeneration, and, unexpectedly, four-repeat tauopathy reminiscent of progressive supranuclear palsy. This peculiar combination of characteristic lesions among neurons, oligodendrocytes, and macrophages, and tau accumulation suggests that the molecular pathology of SCA34 is distinct and pleiotropic. Furthermore, the findings might provide important clues to understand how mutations in a series of elongase genes lead to neurodegeneration.

The most prominent neurological deficit in SCA34 was ataxia [12, 35]; one of the remarkable radiological findings in the brain MRI of SCA34 patients, including this case, is atrophy in the pontine base and cerebellum and the hot cross bun sign in the pons. In this patient, neuronal loss and destruction of transverse fibers (pontocerebellar tract) in the pontine base may explain the symptoms and signs of ataxia and the radiological features. These neuropathological findings were notably similar to other cerebellar ataxias that have pontine base atrophy and the hot cross bun sign, such as cerebellar type of multiple system atrophy (MSA-c) and spinocerebellar ataxia type 2 [20]. However, the CD68 positive macrophages laden with PAS-positive material in the pontine base pathology of SCA34 was not described in MSA-c or spinocerebellar ataxia type 2. The cardinal macroscopic neuropathological findings of MSA-c include atrophy of the pontine base, middle cerebellar peduncles, inferior olivary nucleus, and pontocerebellar fibers representing as cruciform demyelination in the pons and white matter atrophy in the cerebellum. Additionally, depigmentation in the substantia nigra and variable discoloration and atrophy in the putamen can also be found macroscopically [29]. In this study, these findings except for atrophy in the putamen and inferior olivary nucleus were observed in SCA34. And we did not notice glial cytoplasmic inclusions, a hallmark of MSA, or any other abnormal inclusions by anti-alpha-synuclein staining. Nevertheless, MSA may originate from abnormal lipid metabolism [6]. Because distinct involvement of the pontine base neurons and pontocerebellar fibers are common to both SCA34 and MSA-c, these diseases might share a common pathway involving aberrant lipid metabolism. SCA38, a spinocerebellar ataxia caused by mutations in *ELOVL5*, exhibits cerebellar atrophy but no abnormality in the brainstem on MRI [7]. Because the ELOVL5 protein is also a fatty acid elongating enzyme but has non-overlapping substrate specificity and functions as well as catalyzes elongation of shorter carbon chain (between 18 and 22) fatty acids [14], further studies are needed to explain how mutations in *ELOVL4* and *ELOVL5* lead to distinct spatial patterns of neurodegeneration, by analyzing elongases and lipids in the brain. A recent report indicated that a rat model of SCA34 with c.736T>G, p.W246G mutation had a motor impairment and synaptic dysfunction but without overt neurodegeneration in the cerebellar cortex [30]. This rodent model harbors significant similarities to our case and would be useful for further investigations.

When we review aged cases of other SCAs to compare neuropathological characteristics with this SCA34 case, in a rare SCA2 case of 85 years old [23], for example, neuropathological features consistent with SCA2, such as neuronal loss and gliosis distinct in the pontine nucleus, inferior olivary nucleus, cerebellar cortex, posterior column nuclei, substantia nigra, dentate nucleus of the cerebellum, were reportedly observed combined with diffuse neurofibrillary tangles and senile plaques, which are compatible with Alzheimer’s disease pathology (Braak NFT stage IV). Additionally, in an SCA7 case of a 77 year old patient harboring very short 39 CAG repeats in the mutated allele of the SCA7 gene [38], neuropathological changes in cerebral, cerebellar, and brainstem regions were more widespread than in previously reported SCA7 cases with longer repeats and of younger-onset. However, this case only indicated NFT stage I of Alzheimer’s disease pathology. And in an 87 year old autopsy case of SCA31 [43], which is one of the purely cerebellar forms of SCAs and generally prolonged clinical course, comorbid with argyrophilic grain disease, which is also one of the frequent dementing diseases [29], exhibited cerebellar degeneration consistent with SCA31 combined with argyrophilic grains. This case had NFT stage II of neurofibrillary tangles and senile plaques in the neocortex, parahippocampal gyrus, and precentral gyrus and very few alpha-synuclein-positive deposits. Thus, neuropathological findings significantly differ between SCA types and individual cases, and comorbid neuropathological changes related to aging can exist, rather independently of the pathological severity of SCAs. And typical PSP has a much faster clinical course (median survival time 5.6 years in one study) compared to our case [28], although there is a report of an extremely rare autopsy case of PSP with an unusually slow and prolonged disease course (24 years) [24], where a 79 year old woman exhibited widespread neurodegeneration and tauopathy in typically involved regions, and Braak stage III NFTs, senile plaques, but without glial cytoplasmic inclusion. Pathological changes related to PSP were much milder in our SCA34 case compared with the case.

Macrophages, which were CD68 positive and laden with PAS-positive material in the pontine base, were reminiscent of X-linked adrenoleukodystrophy (X-ALD), although we were unable to examine the cleft-like trilamellar cytoplasmic inclusions, which are found in perivascular macrophages in X-ALD, because of our specimen limitation [32]. These macrophages may contain myelin remnants, as suggested in X-ALD [46], considering the marked destruction of myelin in the pontocerebellar fibers of the pontine base. In the current case, we can raise several possible explanations of how *ELOVL4* gene mutations resulted in the generation of such phagocytosed materials in macrophages. First, due to the missense mutation in *ELOVL4,* myelin content might be abnormal and difficult to digest within macrophages. Second, the *ELOVL4* mutation might disrupt organelle functions within macrophages, thus affecting the metabolism of myelin. Third, ongoing massive myelin degeneration might simply surpass the myelin digestion capacity of macrophages. This might be similar, though milder, to the demyelination plaques of multiple sclerosis, where infiltration of massive macrophages laden with myelin is typically seen [37]. Further studies are warranted to clarify the altered function of the markedly abnormal pontine macrophages in SCA34.

Another remarkable finding in this neuropathological study is the frequent vacuoles in the white matter, predominantly in the supratentorial regions. These vacuoles did not abnormally store lipids but were compatible with remnants of degenerated oligodendrocytes, based on anti-Olig2 immunohistochemical and ultrastructural observations. In addition, myelin remnants revealed under the electron microscope also suggests abnormalities in myelin integrity and composition. Although a detailed analysis of lipid metabolism abnormalities is lacking, one plausible explanation for the degenerated myelin in SCA34 is an abnormal or reduced synthesis of complex lipid substances, such as phospholipids and glycolipids, resulting in myelin destruction. Knockout mice genetically lacking ceramide synthase 2 (CerS2) developed vacuoles in the white matter due to myelin ballooning [5, 22]. Curiously, CerS2 interacts with ELOVL4 and other elongase proteins in cultured cells [33]. Mitochondrial encephalopathies, such as Kearns-Sayre syndrome (KSS) [34], also exhibit vacuoles in the white and gray matter. One neuropathological study of KSS suggested that intracytoplasmic oligodendroglial vacuoles of the white matter could be the structural origin of the vacuoles in KSS [41]. The vacuoles in KSS morphologically resemble the vacuoles in our study in that the oligodendroglial nuclei reside within or in contact with single or multiloculated vacuoles and the rims surrounding the vacuoles are positive for MBP. Therefore, a common cascade may cause vacuole formation from oligodendroglial degeneration in both the mitochondrial disease and SCA34.

A previous study in mice showed that ELOVL4 proteins are primarily expressed in neurons throughout the brain and in white matter oligodendrocytes [40]. Thus, it is surprising that only pontine base neurons were severely reduced, whereas other neurons were largely spared in this case. For example, the cerebellar granular layer, which was highly positive by anti-ELOVL4 antibodies in rodents in previous reports [30, 40], was surprisingly preserved in this study. Of note, pontocerebellar fibers, which were most severely affected, showed almost no vacuoles. In contrast, the vacuoles were widespread in the supratentorial white matter of the brain. Thus, cell type- and location-specific metabolic components may be important in the subsequent morphological consequences. In fact, lipid compositions differ from site to site in the brain [31]. Therefore, a more detailed study on lipid metabolism is necessary to fully understand the metabolic alterations in the SCA34 brain. Such future studies may include biochemical analyses for very long-chain fatty acids and perhaps glycolipids in the brain.

Surprisingly, PSP-like pathology was observed in the SCA34 case. When we retrospectively looked back at neurological signs of this case, vertically dominant gaze palsy and trivial akinesia could be clinical features potentially suggestive of PSP. Four repeat tauopathy exhibiting tuft-shaped astrocytes, coiled bodies, and neurofibrillary tangles and pretangles, which are distinctive neuropathological characteristics of PSP, were observed in the regions typically affected in PSP, such as the subthalamic nucleus, substantia nigra, putamen, globus pallidus, and the frontal cortex, although the extent of neuronal loss was none to very mild in those regions. Other typically involved areas, such as midbrain tegmentum, locus ceruleus, red nucleus, nuclei of III, IV, X, XII cranial nerves, and dentate nucleus of the cerebellum were generally preserved. On the one hand, PSP in the current case may be an incidental co-occurrence. A previous study reported that neuropathological features compatible with PSP were found in 45 cases with and 5 cases without clinically diagnosed PSP out of 706 autopsied elderly patients analyzed [16]. Also, in serial forensic autopsies of 998 Japanese cases [48], 4.6% of cases over 60 years old were neuropathologically diagnosed with PSP. Thus, PSP is not an extremely rare entity in the elderly. On the other hand, the four-repeat tauopathy compatible with PSP in the current case may have been caused by the *ELOVL4* mutation. In the literature, at least 19 genetic disorders other than *MAPT*-associated ones displayed tauopathies [42]. At least three cases show some, if not all, of the important neuropathological features of PSP. A case with hereditary Parkinson’s disease/Park8 caused by the *LRRK2* mutation (p.R1441C) showed PSP-like tuft-shaped astrocytic lesions but without the density and distribution diagnostic of PSP [50]. Another case with semantic dementia harboring the *TARDBP* mutation (p.Ile383Val) showed tau-positive astrocytic lesions in the amygdala but not in the subthalamus or basal ganglia, which are usually involved in PSP [17]. One case with the heterozygous *PRKN* mutation (p.C212Y) showed hyperphosphorylated tau lesions consisting of globose-type neurofibrillary tangles and tuft-shaped astrocytes, with distribution consistent with PSP, whereas other family members harboring this mutation (p.C212Y) and another *PRKN* mutation in the compound heterozygous state suffered juvenile-onset Parkinson’s disease [39]. Thus, hereditary disorders that display PSP-like neuropathological features have been reported, although they are very rare. Although PSP is caused by primary alteration of the tau protein, PSP-like deposition of tau may be programmed in the brain as a template that is triggered by aging or other genetic abnormalities like those listed above. We reviewed the exome-sequencing data of this patient in our previous genetic study [35] and discovered no mutations in *MAPT*, *PRKN*, *TARDBP*, and *LRRK2*. Furthermore, aberrant interactions of alpha-synuclein and phosphorylated tau with lipids in a specific subset of neurotransmitter-containing vesicles were described in aging mouse brain [11]; thus, *ELOVL4* mutations may promote tauopathy through abnormal lipid content. Further investigation is needed to determine whether *ELOVL4* mutations can cause PSP-like pathology.

One of the most critical limitations in this study is that this report only describes a single case. The observed neuropathological features should be considered with caution before more autopsied cases of SCA34 are accumulated. When considering the background medical conditions of this patient, the age of 83 years old is an inevitably essential factor that can affect brain neuropathology. For example, white matter volume in the cerebrum decreases with age [29]. This may explain the white matter volume decrease in the current case, at least in part. Among specific neurodegenerative processes that arise with age, Alzheimer’s disease and related disorders are frequent and essential from the viewpoint of tauopathy. In this study, neurofibrillary tangles positively stained with three and four repeat tau antibodies were observed in the limbic system. However, only trace amounts of senile plaques existed, and neuronal loss was not evident in the areas. Thus, these neuropathological changes can be regarded as the early phase of Alzheimer’s disease or a recently proposed entity, primary age-related tauopathy (PART), which exhibits marked neurofibrillary tangles compatible with Alzheimer’s disease in the hippocampus and medial temporal lobes in the absence of amyloid plaques, especially in the elderly [13]. And for tauopathy, subpial astrocytic lesions called thorn-shaped astrocytes along lateral ventricles are often observed in the elderly, and were remarkably observed in this study. However, these age-related changes or diseases cannot explain the currently described four repeat tauopathy or pontine atrophy in the current case. And for alpha-synucleinopathies, such as Parkinson’s disease, Lewy body disease, and multiple system atrophy, we found no evidence of alpha-synuclein accumulation by immunohistochemistry. Potential neuropathological changes caused by atherosclerosis that progresses with age include dilatation of perivascular spaces, microinfarcts, and changes of vessel walls, and white matter changes around vessels such as myelin loss and astrocytosis [29]. Oligodendroglial degeneration and resulting vacuoles are very distinctive lesions that cannot be explained only by atherosclerosis and aging. Myelin degeneration could be influenced by such factors, and requires further investigations in accumulated cases with SCA34 in the future. Finally, kidney failure with a uremic state can accompany neuropathological changes with the presence of Alzheimer type II astrocytes and possibly perivascular neuronal degeneration and demyelination [29]. These changes were not observed in this case, and this medical condition is unlinked to the widespread oligodendroglial degeneration. However, we have to be careful until more autopsied cases with SCA34 are accumulated, and effects by the above-mentioned conditions are considered in those cases in the future.

A recent report described psychomotor deficits in SCA34 patients with another mutation (c.504G>C, p.L168F) [4]. The widespread white matter vacuoles and macroscopic atrophy, especially in frontal, temporal, and parietal lobes, might be associated with the psychiatric symptoms.

We describe the first autopsy case of SCA34 caused by an *ELOVL4* mutation, showing remarkable neuronal loss and pontocerebellar fiber loss in the pontine base. These findings are compatible with the clinical and radiological features in this patient. We also unexpectedly found an accumulation of macrophages laden with PAS-positive material in the pontine base and oligodendroglial degeneration with widespread vacuoles in the white matter. Four-repeat tauopathy that displayed neuronal and glial fibrillary tangles reminiscent of PSP was observed, although whether the tauopathy is coincidental or caused by the *ELOVL4* mutation is unknown. Taken together, these findings suggest that the *ELOVL4* mutation led to neuronal and glial degeneration through pleiotropic pathways in a cell type- and location-specific manner in SCA34.

## Supplementary Information

**Additional file 1**: Additional characterization of the pontine pathology in SCA34. Description of data: Contains additional images and legends, describing additional staining of the pontine pathology in SCA34.

**Additional file 2**: Description of data: Contains additional images and legends, describing abnormal vacuoles in the brain and spinal cord of the SCA34 patient.

## Data Availability

The datasets used and/or analyzed during the current study are available from the corresponding author on reasonable request and with permission of the patient’s family and the institutional review boards, if applicable.

## Abbreviations

HE: Hematoxylin-Eosin
KSS: Kearns-Sayre syndrome
KB: Klüver-Barrera
MRI: Magnetic resonance imaging
MSA-c: Cerebellar type of multiple system atrophy
MBP: Myelin basic protein
PAS: Periodic acid-Schiff
PSP: Progressive supranuclear palsy
SCA: Spinocerebellar ataxia
X-ALD: X-linked adrenoleukodystrophy
