# Supplementary material for: Neuropathology of SCA34 showing widespread oligodendroglial pathology with vacuolar white matter degeneration: a case study
Source: Acta Neuropathol Commun. 2021 Oct 24;9:172. doi: 10.1186/s40478-021-01272-w (PMC8543940; doi:10.1186/s40478-021-01272-w)
Supplement: Supplementary file 1 — Additional file 1: Additional characterization of the pontine pathology in SCA34. Description of data: Contains additional images and legends, describing additional staining of the pontine pathology in SCA34. [file 40478_2021_1272_MOESM1_ESM.pdf]

Supplementary Fig. 1

Additional characterization of the pontine pathology in SCA34

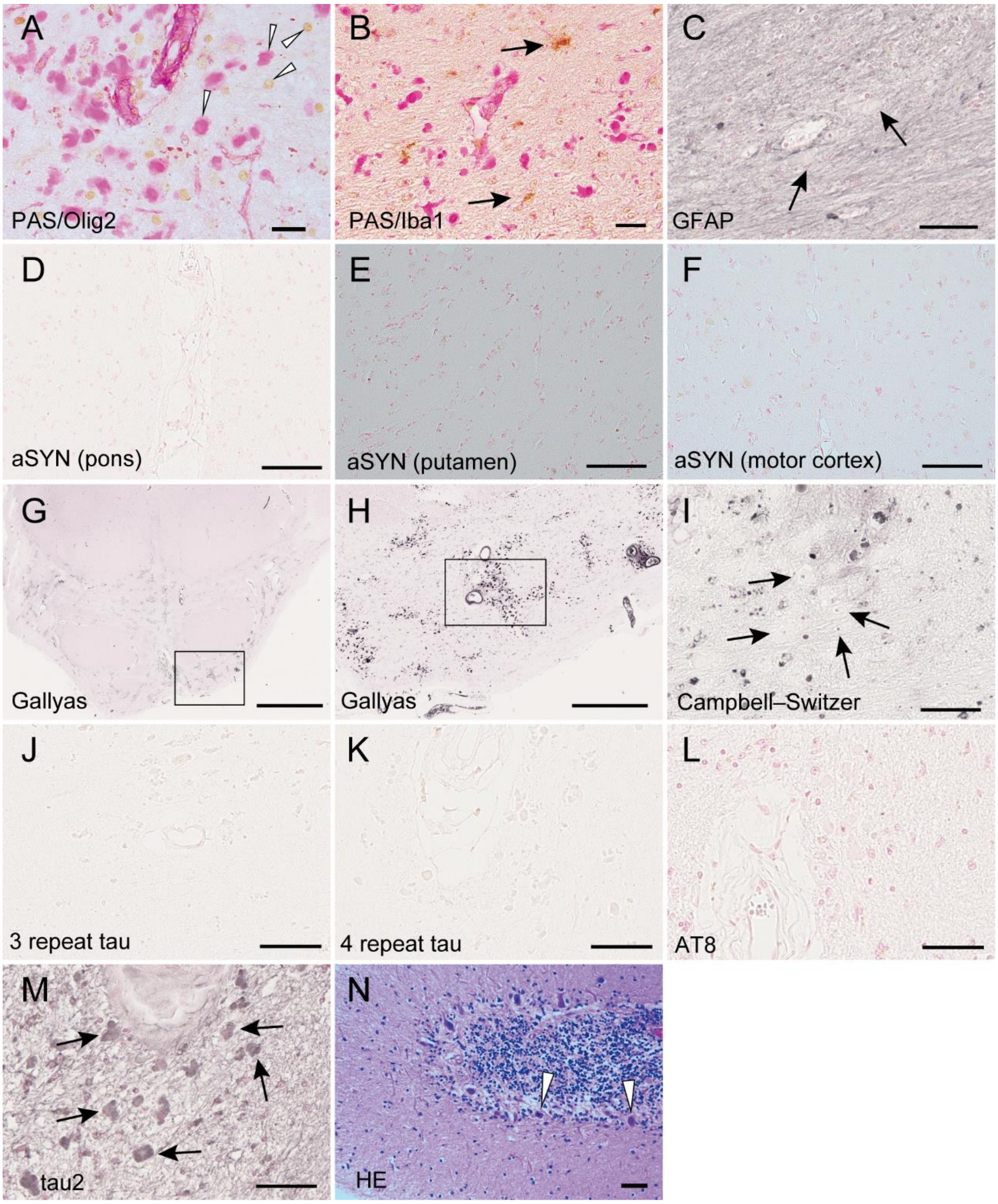

A) Double staining with PAS (red) and anti-Olig2 (yellow) immunostaining showed cells either positive for PAS or Olig2 alone (arrowheads).

B) Double staining with PAS (red) and anti-Iba1 (brown) immunostaining showed that faintly stained cells (arrows) by anti-Iba1 antibody were negative for PAS staining.

C) Anti-glial fibrillary acidic protein (GFAP) immunostaining was negative in the

macrophages (arrows)

D, E, and F) Anti-alpha synuclein (aSYN) staining was negative in the pons (D), basal ganglia including putamen (E), and motor cortex (F).

G and H) Argyrophilia on Gallyas silver impregnation method was exhibited in widespread macrophages in the pontine base excluding pyramidal tracts. H is a magnified view of the rectangle in G. Figure 2K is a magnified view from the rectangle in H.

I) The macrophages did not exhibit argyrophilia on Campbell–Switzer silver impregnation method (arrows).

J, K, and L) Anti-three repeat (J), four repeat (K), and phosphorylated (L) tau were negative in the pontine macrophages.

M) Tau2 immunostaining was mild to moderately positive in the pontine macrophages (arrows).

N) Hematoxylin–Eosin (HE) staining of the cerebellum showed reduced and atrophied Purkinje cells (arrowheads).

Scale bar = 2.5 mm (G), 500 μm (H), 100 μm (D, E, and F), 50 μm (C, I, J, K, L, M, and N), 20 μm (A and B).
