# Supplementary material for: Neuropathology of SCA34 showing widespread oligodendroglial pathology with vacuolar white matter degeneration: a case study
Source: Acta Neuropathol Commun. 2021 Oct 24;9:172. doi: 10.1186/s40478-021-01272-w (PMC8543940; doi:10.1186/s40478-021-01272-w)
Supplement: Supplementary file 2 — Additional file 2: Description of data: Contains additional images and legends, describing abnormal vacuoles in the brain and spinal cord of the SCA34 patient. [file 40478_2021_1272_MOESM2_ESM.pdf]

Supplementary Fig. 2

Vacuoles in the brain and spinal cord of the SCA34 patient

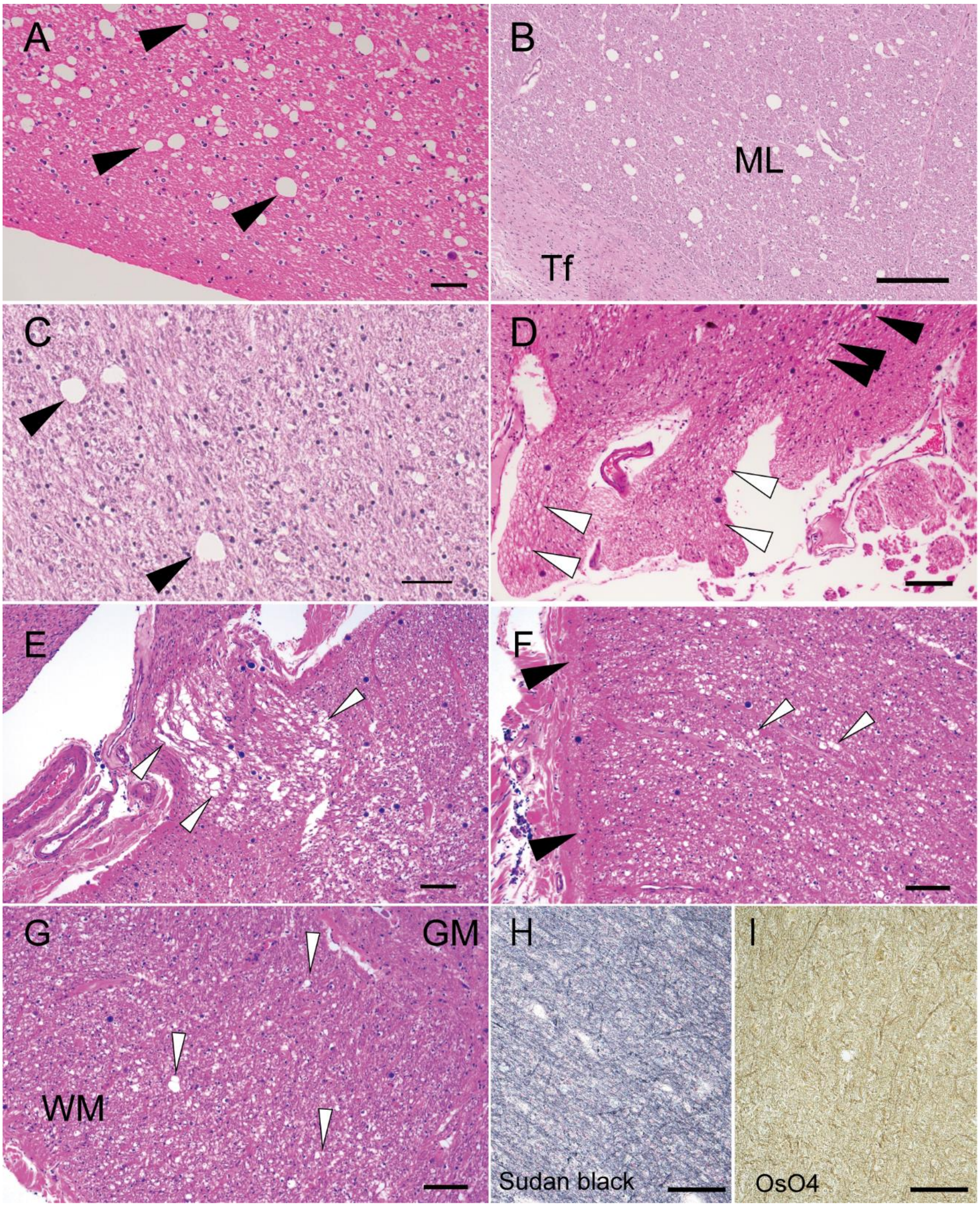

A) Hematoxylin-Eosin (HE) staining of the optic tract showed massive vacuoles (arrowheads).

B) HE staining of the pons showing vacuoles in the right pontine medial lemniscus (designated as ML). Tf indicates the area of transverse pontine fibers.

C) HE staining of the pons showing a very small number of vacuoles in the pyramidal tract (arrowheads).

D) Vacuoles in the right ophthalmic nerve root (white arrowheads) and intramedullary fibers (black arrowheads) of the midbrain stained with HE.

E) HE staining of the lumbar spinal cord revealed vacuoles in bilateral intra-axial posterior roots (the right side is shown) (white arrowheads). In contrast, vacuoles were not observed in the extra-axial root (not shown).

F) The right lateral column of HE-stained lumbar spinal cord showed fibrosis (black arrowheads) and vacuoles along neural fibers (white arrowheads).

G) The right anterior column of HE-stained lumbar spinal cord showed fewer number and smaller sizes of vacuoles (white arrowheads) along the intramedullary anterior spinal root. Gliosis and fibrosis were observed in the anterior horn gray matter (GM).

H and I) The vacuoles in the frontal white matter were not stained with Sudan black (H) and OsO_4 (I), respectively.

Scale bar = 50 μm (A), 250 μm (B), 100 μm (C, D, E, F, G, H, and I).
